# Mindfulness Intervention Improves Coping and Perceptions of Children’s Behavior among Families with Elevated Risk

**DOI:** 10.3390/ijerph20237092

**Published:** 2023-11-21

**Authors:** Jill T. Krause, Samantha M. Brown

**Affiliations:** Department of Human Development & Family Studies, Colorado State University, 1570 Campus Delivery, Fort Collins, CO 80523-1570, USA

**Keywords:** children’s behavior, cognitive coping, mindfulness-based intervention, parenting, underrepresented families

## Abstract

Mindfulness-informed interventions (MIIs) are increasingly common but have not been extensively studied among families with elevated levels of risk (e.g., those involved in child protective services and/or receiving financial assistance). These families often experience high rates of stressors that can impact coping strategies, interpersonal dynamics, and relationships. Given that mindfulness has been shown to promote health and wellbeing, this study used a sample from two pilot randomized controlled trials to test the extent to which a mindfulness-informed intervention improved coping strategies and perceptions of children’s behavior among 53 families with elevated risk. A principal components analysis with a direct oblimin rotation revealed that cognitive–emotion coping strategies could be characterized by three factors: *positive adaptation*, *negative adaptation*, and *positive refocusing*. Intention-to-treat analysis indicated significant group by time differences, with intervention participants demonstrating improvements in positive refocusing coping, positive adaptation coping, and perceptions of children’s behavior problems compared to participants in the waitlist control group. No significant differences were found for negative adaptation coping strategies. Findings provide preliminary support for the benefits of mindfulness training in a sample generally underrepresented in the mindfulness intervention literature.

## 1. Introduction

The proliferation of mindfulness research in recent years has led to an abundance of mindfulness-informed interventions (MIIs [1,2,3,4]), including those specifically targeting parents (e.g., [5,6]; see [7] for a review of MIIs, parenting stress, and youth outcomes). Mindfulness, or the ability to pay attention to the present moment with nonjudgment and curiosity [8], has been associated with lower rates of depression, rumination, and anxiety [9], increased persistence when facing adversity [10], and measures of inhibition and working memory [11]. Mindfulness interventions designed specifically for parents often examine children’s behavioral outcomes and show significant improvements post-intervention [12,13,14]. Indeed, studies show that mindfulness is significantly associated with lower levels of both internalizing and externalizing behaviors in children across developmental stages [15,16]. Children’s behavior problems are a risk factor for later health and development problems, such as lower academic achievement [17] and deficits in self-regulation [18,19]. In addition, they are associated with increases in parenting stress [20]. Several studies have found evidence that behavioral problems persist over time due to a reciprocal relationship between parental stress and children’s behavior [21,22]. Therefore, child behavior problems may indicate the onset or presence of a negative developmental cascade. Certain characteristics of parents, such as their ability to remain mindful, may better allow them to cope with stress, thus permitting them to better respond to their children and interrupt negative relational cycles. In other words, parental mindfulness may be particularly beneficial as a stress buffer with spillover effects that influence children’s behavior.

Despite these findings, few studies have considered the processes by which parental mindfulness and children’s behavior problems are associated [23,24]. Even fewer have evaluated the association in the context of families with elevated levels of risk, who are typically underrepresented in mindfulness research and may be particularly vulnerable to stress [25,26], making these families poised to benefit from MIIs. One potential key benefit of MIIs may be improvements in adaptive coping. Parents who participate in an MII are able to use more positively oriented or adaptive coping strategies that inhibit stress from disrupting their parenting and, therefore, allow them to view their child’s behavior more favorably. This may prevent aversive parent–child interactions and result in fewer actual behavior problems. Cognitive coping, or the mental strategies an individual employs after a negative event to regulate their emotions or behavior [27,28], may therefore play a critical role in the association between parents’ mindfulness and their children’s behavior. The present study seeks to examine whether an MII influences coping strategies and perceptions of children’s behavior in a sample of families with elevated risk, thus, filling a gap in the field’s understanding of the processes that underlie effective MIIs for underrepresented families.

### 1.1. Stress, Parenting, and Mindfulness

Extant theory, such as the Family Stress Model (FSM) [29] and Belsky’s [30] Determinants of Parenting, points to stress as a key factor influencing parenting. The FSM suggests that a hardship or major stressor disrupts the family and generates pressure, leading to parental psychological distress, interpersonal problems, impaired parenting, and, ultimately, child adjustment issues [29]. Recent empirical evidence from an illustrative review and longitudinal studies supports these claims [29,31,32]. Belsky’s [30] model posits that three domains are major determinants of parenting behaviors, suggesting possible targets for interrupting the pathway between stress and disrupted parenting: the parent’s psychological resources, the child’s characteristics, and the parent’s sources of stress and support. Therefore, stressors may impact parenting directly (e.g., through needing to work additional shifts for financial reasons), or indirectly (e.g., through causing psychological distress beyond the parent’s psychological resources). The model proposes a hierarchy for the importance of these influences on parenting behaviors, with the parent’s personal resources as most influential, followed by contextual supports or stressors, and, lastly, the child’s characteristics [30]. As with the FSM, recent reviews and studies have supported Belsky’s model of parenting [33,34]. Together, these two theories convey that a parent’s psychological resources are critical in disrupting the pathway between a stressor and its effect on parenting behaviors and child behavior problems.

Coercion theory [35] explicitly lays out how negative parenting can create a coercive cycle between parent and child. Those who are unable to effectively respond to problem behavior often escalate aversive interactions and reinforce the problem behaviors, thus creating a cycle in which, over time, children grow increasingly aggressive, and parents lose control in handling the situation. In the forty years since coercion theory was proposed, the model has become central in explaining failures in coregulation as it has amassed empirical support [36] Therefore, a key intervention target is to prevent negative parent–child interactions by bolstering parents’ ability to handle stress and children’s challenging behavior. 

Given how pernicious the effects of stress can be and the ways in which stress spills over into family functioning, more attention should be paid to populations experiencing elevated levels of risk that may increase stress or affect the stress response, such as involvement with human services, being in a single-parent home or otherwise socially isolated, living in poverty, or having substance-use issues [37,38,39]. These families have largely been absent from intervention research, especially in the context of mindfulness, and are at greater risk of poor outcomes, such as child maltreatment [40], intimate-partner violence [41], and mental health problems [42]. Families with elevated risk may particularly benefit from tailored interventions because risk factors tend to be bundled together [43], and greater risk is associated with greater levels of stress [44,45]. Therefore, families with elevated levels of risk may have greater impairments in parenting behaviors. 

Mindfulness may be a key psychological resource for parents. Mindfulness has been associated with many benefits, among which include improved emotion regulation capabilities, increased psychological flexibility, and lowered perceptions of stress [46,47]. The mindfulness stress buffering hypothesis [48] proposes that mindfulness training can reduce perceptions of stress and in turn reduce the body’s heightened response to stress. Tests of this hypothesis have been promising [49,50]. Mindfulness can serve as an effective buffer against stressors both proximal, such as coping with substance use cravings [51] or mental fatigue from multitasking [52], and distal, by reducing perceptions of and reactions to stress over time [48]. Short-term MIIs with parents have been shown to increase levels of mindfulness and subjective wellbeing, decrease levels of parenting stress, and improve youth outcomes, such as externalizing behaviors [5,7,53]. Results from the few studies utilizing underrepresented samples suggest that MIIs are feasible and acceptable [6,54,55] and they are often cost-effective to administer [56]. Therefore, brief MIIs for families with elevated levels of risk may be cost-effective to implement and provide critical tools for parenting effectively in the face of stress and children’s challenging behavior. 

Beyond consistent links between mindfulness and personal wellbeing, there is evidence that greater parental mindfulness is related to better outcomes for their children, and, thus, may have spillover effects in the family. For instance, in a cross-sectional study of parents with children ages 3 to 17, parents’ dispositional mindfulness was associated with fewer internalizing and externalizing symptoms, an association that operated indirectly through higher levels of mindful parenting and lower levels of negative parenting practices [15]. A recent meta-analysis of MIIs complements these findings, noting that participation in mindfulness training was associated with improved youth outcomes [7]. Short-term MIIs for parents have led to improvements in children’s behaviors, quality of life, executive functioning, and psychological distress [7,55,57]. 

This pattern of findings supports the assumption that parents who are mindful or those who are trained in mindfulness may be better equipped to regulate their own emotions and respond sensitively to the emotional cues and needs of their children. They may be less reactive to stress and more likely to practice self-compassion, serving as an effective model of how to respond to life’s inevitable stressors. It follows that when faced with challenging behavior from their children, mindful parents may better be able to deescalate situations rather than engaging in a coercive cycle. These parents may also be less likely to exhibit reactive parenting when faced with stressors unrelated to their child. Though these findings provide a strong rationale for future research into the relationship between mindfulness, parenting, and children’s outcomes, further process-oriented research is needed to determine how mindfulness leads to improvements in family systems.

### 1.2. The Role of Coping

One process that may play a key role in how mindfulness acts as a stress buffer is through adaptive coping strategies. Adaptive coping strategies are those that are effective in reducing or mitigating stress, leading to improved outcomes [58]. Broadly, an individual’s cognitive appraisal of a situation impacts the initiation and choice of coping efforts [59], as well as their ensuing emotional response [60,61]. By teaching parents to remain nonjudgmental and grounded, mindfulness may support more adaptive cognitive appraisals of stressors, including how they perceive their children’s behavior. There is empirical evidence to support these theoretical predictions. For example, in a longitudinal study of participants who participated in a 7-week mindfulness-based course, participants who received the mindfulness training showed increases in problem-focused coping that were associated with increases in wellbeing, despite low levels of formal mindfulness practice [62]. Other studies have found that short-term MIIs lead to improvements in coping flexibility and adaptive coping, decreases in maladaptive coping, and decreases in negative parent–child interactions [63,64], and that mindfulness training increases the ability of individuals to self-monitor and select appropriate coping strategies in times of stress [65]. Studies of trait mindfulness (i.e., dispositional mindfulness [66]), which refers to the innate capacity to remain mindful in everyday life, have yielded similar findings; one study in adults found that awareness of the present moment was positively associated with adaptive coping strategies [67]. As such, mindfulness may be associated with more adaptive coping as it promotes present moment awareness, self-compassion, and nonjudgment, thereby influencing the cognitive appraisal of and response to a potentially stressful situation. 

Though coping processes are not inherently good or bad and should be evaluated within the time and context in which they occur [59,60,68], they are often broadly categorized as either adaptive (e.g., positively reappraising situations for the better, planning for the future) or maladaptive (e.g., ruminating on prior experience, catastrophizing), and this bifurcated terminology is frequent in the literature. For example, adaptive coping processes have been associated with lower depressive symptoms [69] and maladaptive coping with greater distress and burnout in parents of children with autism [70]. Despite potential flaws of this dichotomy, broader analysis of coping beyond the specific strategy or individual subscale may be useful in determining what patterns there are across facets of coping and for which individuals. 

The positive appraisal style theory of resilience [60,61] posits that how an individual typically responds to stress and aversive stimuli is a key influence in their resilience and mental health. Some studies have grouped subscales of coping measures into two categories and created a composite score for adaptive or maladaptive coping [71,72]. At least one study has used confirmatory factor analysis with eight of the nine subscales on the Cognitive Emotion Regulation Questionnaire (CERQ) to identify a bifactor pattern in the data, resulting in an adaptive and maladaptive factor [73]. In other words, though coping is time- and context-dependent, individuals may have a typical response to stress that plays an important role in their resilience and mental health. Indeed, positive or more adaptive styles of coping may promote parental resilience and psychological wellbeing, and therefore have a positive influence on perceptions of children’s behavior. Through promoting awareness, self-compassion, and nonjudgment, MIIs support the adoption of more adaptive coping patterns and can bolster family functioning. 

In sum, families with elevated levels of risk may be particularly vulnerable to maladaptive coping patterns. Higher risk is associated with higher levels of stress [25,26], and high levels of stress have been associated with maladaptive coping, which in turn increases, rather than decreases, stress levels [74]. This transactional nature of coping has been further theorized to influence children’s development of coping strategies; parents serve as a role model for their child, and the child’s subsequent coping serves as an active ingredient in risk, resilience, and various developmental cascades [75]. In other words, parents in families with elevated risk may be vulnerable to engaging in maladaptive coping due to a pileup of stressors, thereby influencing their children in adopting maladaptive coping strategies. As such, providing these families with tools, such as mindfulness, may prevent parents from engaging in coping strategies that are harmful to their wellbeing and that of their family and encourage them to perceive stressors in a less problematic way. 

### 1.3. Coping and Children’s Behavior Problems

Two processes by which positive cognitive appraisals of stressors or aversive stimuli can promote psychological wellbeing are by (1) reappraising or reframing situations and (2) inhibiting negative appraisals and the emotional reactions that coincide with them [60,61]. The implication is that for parents, positive appraisals promote their mental health and prevent aversive parent–child interactions by inhibiting mutual escalation in times of stress. Thus, positive appraisals can potentially reduce harsh parenting. According to the positive appraisal style theory of resilience, positive subjective cognitive appraisals are the proximal mediator for resilience in that cognitive appraisals precede emotional responses [60,61]. That is, parental cognitive coping strategies directly influence their emotional responses to their children and, thus, influence their interactions and children’s outcomes for the better or worse. Dix’s [76] work on the role of parent attributions and children’s development is in line with this assertion; Dix posits that a parent’s reasoning for their children’s behavior influences their interactions with them, which in turn influences their children’s view of themselves and how they should act. Empirical evidence has been largely supportive of these claims [77]. Relatedly, the Double ABC-X model [78] places emphasis on how the parent’s or family’s understanding of events can produce or influence their experiences of events. Empirical tests of this model suggest that as stress or negative events pile up, the family’s ability to cope is influenced by their resources and their perceptions (i.e., cognitive appraisals) of the situation, and, in turn, this influences how well they adapt [79]. Therefore, it follows that parents who cope in more positive or adaptive ways should have children who exhibit fewer behavior problems as an indicator of their adaptation.

### 1.4. Mindfulness-Informed Interventions for Underrepresented Populations

MIIs may be particularly well suited to bolster family functioning, especially in families with elevated risk. In sum, the literature suggests that mindfulness may be a key psychological resource such that MIIs may encourage parents to cope in more adaptive ways and improve perceptions of children’s behavior. Short-term mindfulness interventions have led to improvements in mindfulness, coping, and perceptions of stress and children’s behavior, and are feasible, acceptable, and cost-effective to implement with families with elevated levels of risk [54,56,63,64,65]. MIIs provide tools for self-awareness, self-compassion, stress buffering, and adaptive coping; thus, parents who participate in an MII may be better able to maintain present-moment awareness, regulate their own emotions, and respond sensitively to the emotional cues, needs, and challenging behaviors of their children. They may have more adaptive coping processes buffering them against the deleterious effects of stress as well as improving interactions with their children and perceptions of children’s behavior. Examining these associations in families with elevated risk will provide crucial insight into populations underrepresented in the MII literature.

### 1.5. The Current Study

The present study had dual aims: (1) to determine whether broad patterns of coping would be revealed following an oblimin-rotated principal component analysis (PCA) of a cognitive coping measure, and (2) to examine whether an MII influenced parents’ coping strategies and perceptions of children’s behavior problems in families with elevated levels of risk. It was hypothesized that (a) the rotated PCA would reveal factors indicative of broadly adaptive and broadly maladaptive cognitive coping patterns and (b) the MII would have positive effects on parents’ coping strategies and perceptions of children’s behavior, such that the intervention group would show an increase in adaptive coping strategies and a decrease in negative perceptions of children’s behavior. We found evidence for three broad strategies of coping. The MII increased parent’s use of positive adaptation coping skills and reduced their perceptions of problem behavior in their children Studying these associations in families with elevated risk is imperative as the field looks to make MIIs more accessible to underrepresented populations, as little mindfulness research has been conducted on families with diverse backgrounds and complex needs. 

## 2. Materials and Methods

Data were collected from two pilot randomized controlled trials conducted in the U.S. state of Colorado, testing the effects of an MII for families with elevated risk. Most families were recruited from the Denver metro, an extended urban area that also contains many suburbs. In the first pilot study (cohort 1), families involved with child protective services with co-occurring substance misuse were recruited to examine the feasibility, acceptability, and initial efficacy of the intervention. The second pilot study was implemented using an enhanced version of the mindfulness-informed intervention. Specifically, the intervention was adapted based on participant feedback to better target broad stressors and coping strategies. In addition, recruitment was expanded to be more inclusive of families involved with human service systems with broad experiences of stress rather than solely focusing on families with substance misuse (cohort 2). Across the two cohorts, parents were eligible if they could speak and understand English, as both study measures and intervention materials were normed for and delivered in English. Families were also eligible if the parent lived with their child or had regular, weekly visitation with their child [80].

### 2.1. Participant Characteristics

There were 53 parents who participated in the two pilot studies, with 28 participants in cohort 1 and 25 participants in cohort 2. Most parents were female (90.6%) and were between the ages of 21 and 53 years, with an average age of 31 years (*SD* = 7.5). On average, families had 2.6 (*SD* = 1.4) children in the home. Target children were between 1.5 and 16 years old, with an average age of 5.6 years (*SD* = 4.5), and slightly more than half were male (54.7%). Most participants (64.2%) had no spouse or cohabitating partner. Parents identified racially and ethnically as White (49.1%), followed by Black (20.8%), Latinx (18.9%), biracial (7.5%), American Indian/Alaskan Native (1.9%), or selected “Other” (1.9%). 

About half (49.1%) of the sample was employed. A majority (69.8%) earned 20k USD annually or less, with only 5.7% of participants making 50k USD annually or more. In terms of education, most parents had completed high school or some college (76.4%), 15.1% had less than a high school diploma, and 9.1% were college graduates. Over half of participants (52.8%) had prior involvement with the Colorado Department of Human Services for concerns of child abuse and/or neglect. 

### 2.2. Procedures

A pretest–posttest randomized control group design was used, currently considered the gold standard for evaluating intervention effectiveness [16]. Staff shared the study opportunity with families and were contacted by the research team to confirm eligibility. Eligible families were invited to participate in a home-based interview. Participants provided written informed consent to the research staff (i.e., the second author of this manuscript or a graduate research assistant) prior to completing preassessments and randomization. Families were randomly assigned to receive the six-week MII or the waitlist control group, which received treatment as usual (TAU), which may include ongoing human service system involvement (e.g., financial assistance, home visits, parenting classes). In families with multiple parents or guardians or multiple children, respondents were asked to choose a single target parent and target child to serve as reference points for assessments and participation in the intervention. Families completed post-assessments following the intervention period, approximately 6–8 weeks later, and were compensated 100 USD for their participation. This financial incentive was likely motivating for some participants with lower incomes. Research indicates that although high financial incentives reduce response time [81], results relating to participant outcomes have been mixed. Some studies have found no evidence that incentives alter participant cognitive biases [81], whereas others have found gender-related differences in relation to type of compensation [82]. However, monetary incentives have been associated with greater retention of participants with low incomes [83] and compensating participants to commensurate their time is best-practice. Families were randomized to the intervention on a rolling basis as they were recruited, which resulted in uneven groups. Across the two cohorts, there were 30 families in the intervention group and 23 families in the waitlist control group. 

#### Mindfulness-Informed Intervention

Parents randomized to the intervention condition received six weekly, individualized, and in-home sessions. Across the two cohorts, sessions followed a structured format with four parts. Participants (1) completed a brief check-in and then were provided (2) psychoeducational content focused on stressful experiences, mindfulness, cognitive–emotion regulation, and parenting. This was followed by (3) experiential mindfulness-informed exercises, such that parents were taught both formal (e.g., mindful breathing) and informal (e.g., mindfulness of daily tasks) strategies. Mindfulness exercises were designed to broaden their awareness and acceptance of stressors in addition to strategies to support self-regulation of thoughts, feelings, and emotions in present-moment experiences, including interactions with their children. In the first cohort, session content was tailored toward triggers that may elicit substance use and other related stressors. In the second cohort, session content was tailored toward the use of problem-solving coping strategies in relation to participant-identified stressors. Table 1 outlines general themes by session for each cohort. At the end of each session, (4) parents were asked to reflect and share their experiences of the session content and exercises. This strengths-based intervention ultimately aimed to increase cognitive control over emotional distress and regulate stress-related responses that, when unaddressed, may result in poor parental wellbeing and family dysfunction. 

### 2.3. Measures

#### 2.3.1. Parental Cognitive–Emotion Coping

Parent’s cognitive–emotion coping was measured using the Cognitive and Emotion Regulation Questionnaire Short-Form (CERQ-SF) [84]. Participants were asked to indicate on a scale from 1 (*almost never*) to 5 (*almost always*) how they generally think after experiencing a negative or unpleasant event. In the original version of the CERQ-SF, there are nine subscales comprising two items each (self-blame, acceptance, rumination, positive refocusing, refocus on planning, positive reappraisal, putting into perspective, catastrophizing, and other-blame), with no composite or total score. For the current study, we used seven of the nine subscales (excluding self-blame and other-blame) that we hypothesized to change after participating in the mindfulness intervention. Although prior research has demonstrated relatively strong internal consistency among the CERQ-SF subscales, with Cronbach’s alpha ranging from 0.61 to 0.90 [85,86,87], several subscales demonstrated poor reliability (e.g., Cronbach’s alpha = 0.50 for acceptance, 0.47 for rumination, and 0.54 for putting into perspective) in our sample. To remedy this and examine if there were broader patterns of coping within our sample, an oblimin-rotated principal components analysis (PCA) was computed using the seven subscales, and resulting factor scores were used in our subsequent analyses.

#### 2.3.2. Parental Perceptions of Children’s Behavior Problems

The Child Behavior Checklist (CBCL) [88,89] was used to assess parent perceptions of children’s behavior. This study utilized the preschool forms for children aged 1.5–5 years old and the school-aged forms for children aged 6–18. Both forms asked parents to rate the degree (0 = *not true*, 1 = *sometimes true*, 2 = *very true*) to which a statement (e.g., “demands a lot of attention”, “feels worthless or inferior”, “threatens people”) best describes their children’s emotions or behaviors. The preschool form consists of 99 items and focuses on the child’s behavior now or in the past two months, whereas the school-age form consists of 112 items assessing the child’s behavior now or in the past six months. The creators of the form used normative samples to standardize. The CBCL is among the most well-known and commonly used behavior checklists [90]. Various subscales can be derived from the form, though this study utilized the composite score for total behavior problems. Studies that use total behavior problems consistently report strong reliability, such as a Cronbach’s alpha of 0.95 [91]. Further, total behavior problems have been associated with clinical diagnoses [90]. Therefore, total problems may be a better representation of the parent’s perceptions of their children’s behavior than a specific subscale. The score for total problems, encompassing both internalizing and externalizing problems, was calculated by summing all items of the CBCL, with higher scores indicating more parent-reported behavior problems. Raw scores were then standardized following CBCL scoring guidelines to account for differences due to age and gender.

#### 2.3.3. Covariate

Cohort was included in the model as a covariate for several reasons. First, the two cohorts took place at different points in time, thus controlling for cohort accounts for any sociopolitical variance (i.e., historical effects). Second, several differences between the two cohorts may influence the variables of interest. The inclusion criteria for cohort 1 included evidence of substance misuse, which has been associated with negative parent, child, and family outcomes relevant to the current study [92,93]. Additionally, cohort 2 had explicit goals related to stress-reduction in the context of problem-solving.

### 2.4. Data Analysis 

All analyses were conducted in IBM SPSS Statistics for Windows, version 28 (IBM Corp., Armonk, NY, USA). SPSS has the ability to conduct the type of analyses we sought to complete and is the primary statistical package available for researchers at the authors’ institution. This software and version have been used in previous research with similar aims of evaluating intervention effectiveness [94]. Descriptive statistics were calculated for sample characteristics. Due to the poor internal consistency of some of the CERQ-SF subscales, we conducted principal component analysis (PCA) to examine whether the factor structure of the CERQ-SF differed for our sample [95]. PCA was conducted on 14 items (from the seven selected subscales) from the CERQ-SF, and adequacy of the data and model was assessed using the determinant, the Kaiser–Meyer–Olkin (KMO) measure of sampling adequacy, and Bartlett’s test of sphericity. For the data to be adequate for dimensionality reduction, such as PCA, we would expect a nonzero determinant, a KMO above 0.6, and a statistically significant Bartlett’s test of sphericity [96,97,98]. The scree test was used to determine the number of components to retain [95], which allows for visual examination of a graphical illustration of the eigenvalues. Eigenvalues are illustrated by dots and presented in descending order [99]. A cutoff point is established when the dots level off; thus, the number of components that should be retained is determined by the eigenvalues that are above this cutoff point, typically when eigenvalues > 1. To evaluate the items that loaded strongly onto each component, loadings of 0.55 or greater were considered good [100]. 

To examine change in cognitive coping and perceptions of children’s behavior from pre- to post-assessment, intention-to-treat (ITT) analyses were conducted, using linear mixed models on the entire randomized sample (*N* = 53). ITT analyses are a method in which all randomized participants are included in the model, even if they did not complete the intervention [101]. For RCTs with high ecological validity, ITT is often considered the most rigorous method for determining intervention effects [53]. In our models, treatment group and time were fitted as fixed effects, controlling for cohort, and random intercept was selected for participants. Treatment group was modeled as a categorical variable (intervention vs. waitlist control), time as a repeated measure (pre- and post-assessment), and a group × time interaction term as the primary parameter of interest. All models were estimated with maximum likelihood (ML) methods, and 95% confidence intervals (CIs) were calculated. As a measure of effect size, we computed Hedges’ *g* [102]. Hedges’ *g* adjusts for small sample sizes and any heteroscedasticity. 

## 3. Results

### 3.1. Factor Extraction and Rotation

Prior to interpreting the results of the PCA, we examined whether the data were appropriate for data-reduction techniques. Small samples sometimes have issues with sampling adequacy and may not be well suited for PCA. However, the determinant was 0.003, the KMO measure of sampling adequacy was 0.65, and Bartlett’s test of sphericity was statistically significant (*p* < 0.001), suggesting that the data were sufficient for dimensionality reduction. 

Next, to approach simple structure, a direct oblimin rotation was applied to the PCA, allowing the components to correlate, reflecting potential real-world correlations in coping strategies. The scree test indicated a four-factor solution that accounted for 59.29% of the variance. However, inspection of the pattern matrix indicated that one factor consisted of a single item (“I think that I have to accept that this negative situation has happened”) and one item (“I think that it hasn’t been too bad compared to other things”) negatively loaded on a factor and did not conceptually fit. As such, these items were excluded from analyses, which resulted in a final three-factor solution. For the three-factor solution, the determinant was 0.011, the KMO measure of sampling adequacy was 0.65, and Bartlett’s test of sphericity was statistically significant. The final three-factor solution also explained 61.53% of the variance in participant’s cognitive coping strategies. 

The item-factor loading matrix for the final three-factor solution with the included 12 items from the CERQ-SF is presented in Table 2. Six of these items loaded on factor 1, four items loaded on factor 2, and two items loaded on factor 3, all with loadings of 0.55 or greater. Factor 1 included items from the positive reappraisal, acceptance, putting into perspective, and refocus on planning subscales of the original CERQ-SF and was conceptualized as *positive adaptation coping* (eigenvalue = 3.29, % variance explained = 27.44). Factor 2 included items from the rumination and catastrophizing subscales of the original CERQ-SF and was conceptualized as *negative adaptation coping* (eigenvalue = 2.59, % variance explained = 21.56). Factor 3 consisted of the two items comprising the positive refocusing subscale of the original CERQ-SF and was conceptualized as *positive refocusing coping* (eigenvalue = 1.50, % variance explained = 12.53). Internal consistency for the three factors was also examined using Cronbach’s alpha. Cronbach’s alpha for the individual factors were good: α = 0.76 for *positive adaptation coping*, α = 0.76 for *negative adaptation coping*, and α = 0.70 for *positive refocus coping*. 

### 3.2. Coping Strategies

**Figure 1:**
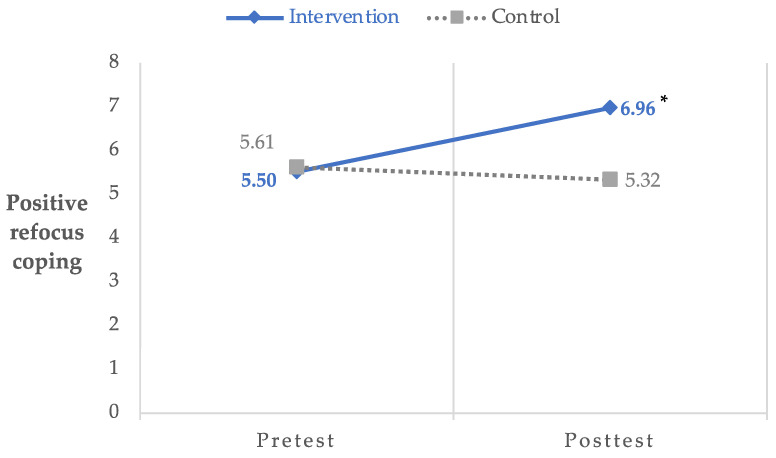
Group by time differences in positive refocus coping. * *p* < 0.01; Hedges’ *g* = 0.87.

**Figure 2:**
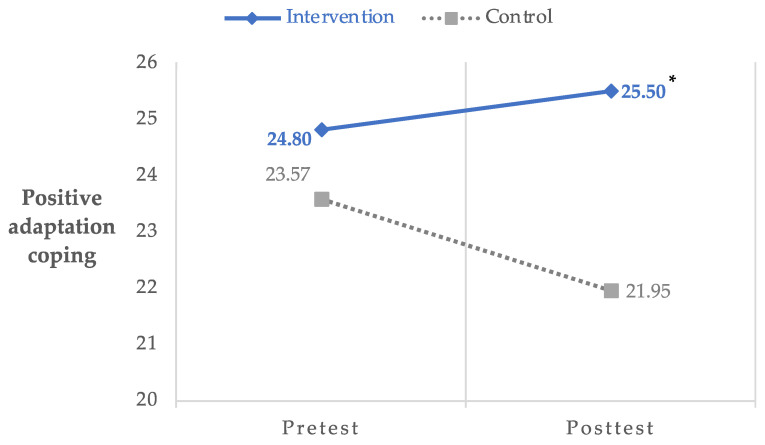
Group by time differences in positive adaptation coping. * *p* = 0.05; Hedges’ *g* = 1.08.

Items were summed for each factor and used in ITT analyses to determine the effects of the MII on cognitive coping strategies. ITT analyses revealed a significant group by time effect for positive refocus coping, *F*(1, 46.62) = 9.72, *p* < 0.01, 95% CI: 0.57–2.65, Hedges’ *g* = 0.87. Specifically, participants in the intervention group reported improvements in positive refocus coping from pre- (*M* = 5.50, *SD* = 2.10) to post- (*M* = 6.96, *SD* = 1.99) assessment compared to participants in the waitlist control group (preassessment: *M* = 5.61, *SD* = 1.80; post-assessment: *M* = 5.32, *SD* = 1.73). In addition, a marginally significant group by time effect was found for positive adaptation coping, *F*(1, 46.18) = 3.94, *p* = 0.05, 95% CI: −0.03–4.65, Hedges’ *g* = 1.08, with the intervention group reporting improvements in positive adaptation coping from pre- (*M* = 24.80, *SD* = 3.95) to post- (*M* = 25.50, *SD* = 3.18) assessment compared to participants in the waitlist control group (preassessment: *M* = 23.57, *SD* = 4.89; post-assessment: *M* = 21.95, *SD* = 3.41). There were no significant group by time effects for negative adaptation coping, *F*(1, 51.85) = 0.05, *p* = 0.83, 95% CI: −3.68–4.58. Figure 1 and Figure 2 chart the significant change in coping strategies reported by the intervention group.

### 3.3. Perceptions of Children’s Behavior Problems

**Figure 3:**
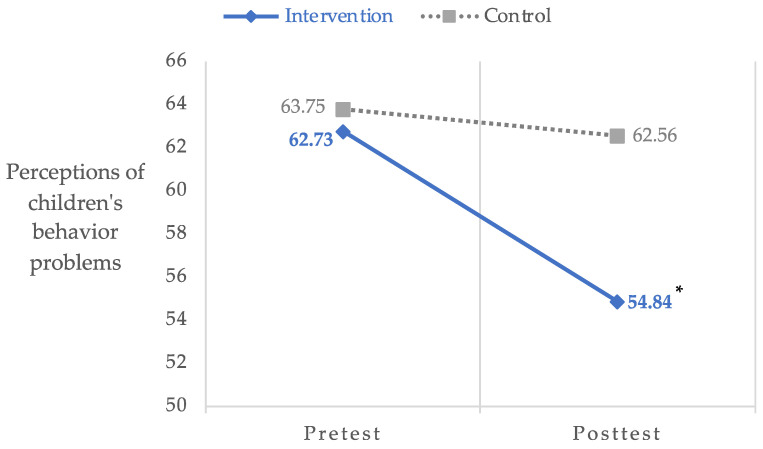
Group by time differences in perceptions of children’s behavior problems. * *p* < 0.01; Hedges’ *g* = 0.66.

ITT analyses revealed significant group by time effects for perceptions of children’s behavior problems, *F*(1, 36.69) = 7.51, *p* < 0.01, 95% CI: −11.42–1.71, Hedges’ *g* = 0.66. Specifically, participants in the intervention group reported decreases in perceptions of children’s behavior problems from pre- (*M* = 62.73, *SD* = 10.83) to post- (*M* = 54.84, *SD* = 11.65) assessment compared to participants in the waitlist control group (preassessment: *M* = 63.75, *SD* = 9.55; post-assessment: *M* = 62.56, *SD* = 7.31). Figure 3 shows this change. 

## 4. Discussion

This study sought to examine patterns of coping and the extent to which an MII changed coping strategies and perceptions of children’s behavior in a sample of families with elevated levels of risk. Consistent with our hypothesis, the rotated PCA yielded interpretable factors: *positive adaptation* and *negative adaptation*, which explained most of the variance in participant responses. This finding aligns with prior research that found patterns of adaptive and maladaptive coping using the same cognitive coping measure [64,73]. We did not make specific hypothesis regarding the *positive refocusing* factor, which replicated the subscale *positive refocusing* on the original measure. We did not anticipate that these items would be unique from other adaptive cognitive processes. However, cognitive restructuring was a central focus of the MII, and thus participants may have acquired specific skill related to this strategy. Broadly, these findings provide limited support for the positive appraisal style theory of resilience [60,61] in that there were some general patterns across which parents employed coping strategies, including a generally adaptive and generally maladaptive pattern. However, given both the strong theory supporting coping as situation- and resource-dependent and the unique dimension of positive refocusing evidenced here, further research should include additional standardized measures of coping to see if our results can be replicated.

In examining outcomes from the MII, the overall effect of the intervention was medium to large (i.e., Hedges *g* = 0.66–1.08), which is common in small-sample studies and will need to be replicated with larger samples. Our hypothesis was supported in that participants in the intervention group demonstrated improvements in adaptive coping in both the *positive refocusing* and *positive adaptation coping* strategies. Mindfulness has been hypothesized as a buffer against stress [48] and emphasizes present moment awareness, nonjudgment, and self-compassion, all of which have been associated with adaptive coping [65,103]. Previous MIIs have found improvements in problem-focused coping, coping flexibility, and general adaptive coping [62,63,64]. Consistent with our findings, this suggests that even relatively short-term mindfulness training can have positive effects on the ways in which parents cope with stress. It is also possible that adaptive coping may decline over time if parents are not provided support, as demonstrated by scores from pre- to post-assessment among control group participants. Because the MII was strengths-based, and the goal was to promote new tools to mitigate stress and improve parent–child relationships, we did not make specific hypotheses regarding the *negative adaptation coping* factor. Indeed, providing brief mindfulness training to parents can lead to more adaptive coping, which may in turn help manage stress and interpersonal relationships. 

The MII also proved effective in decreasing perceptions of children’s behavior problems; those in in the intervention group reported significantly fewer children’s behavior problems at post-assessment. Interventions that target parental mindfulness may be a means to improve how parents respond to children’s behavior and perhaps overall family functioning in families with elevated levels of risk. Given that some MIIs are low-cost and can be delivered flexibly, they may be an ideal intervention strategy for those planning to serve underresourced and underrepresented communities. Mindfulness interventions have now been used effectively with parents for intervention [7] and prevention [12], with clinical and nonclinical populations [104] and welfare-adjacent families [80]. Ultimately, from a public health perspective, this study provides preliminary support that MIIs for those with elevated levels of risk can improve coping and reduce spillover effects, thereby providing crucial to support to families who may not otherwise have the support or resources to thrive. 

### Limitations and Future Directions

Despite several strengths of this study, such as the inclusion of an underrepresented sample to participate in a tailored MII, this study is not without limitations. Tailoring the intervention to meet the unique needs of families with elevated risk was associated with increases in positive adaptation coping and positive refocusing coping and decreases in perceptions of children’s behavior problems. This indicates that despite minor differences in intervention delivery across cohorts, a relatively short MII may benefit these families. However, it may be difficult to identify the precise mechanisms underlying change from pre- to post-assessment or exactly which elements of the intervention were instrumental to its success.

Relatedly, due to the small sample size, we were unable to test whether improvements in perceptions of children’s behavior problems are the result of mindfulness training alone or increases in adaptive coping. It is possible that these parents were generally able to view their child’s behavior as less problematic by remaining mindful and self-compassionate. Another possibility is that in the face of challenging behavior or stress, parents were better able to choose adaptive coping strategies and thus inhibit coercive cycles that may lead to poor parent–child interactions. Despite this limitation, our findings provide at least partial support for spillover effects from mindfulness training. Parent-focused MIIs can yield positive effects for other family members, bolstering family functioning.

Another limitation of the small sample is that it limits our ability to conduct additional analyses. The processes through which mindfulness training may influence family outcomes as well as generalize to other underrepresented populations are still largely unknown, and we were not statistically powered to examine them. In addition, the study solely relied on survey data from one parent about one child, despite their family makeup. Since the intervention was aimed at improving family functioning, failing to include measures from other family members may introduce social desirability bias or may ignore how the MII affected others in the family system. This common-method variance can be addressed in future research by including objective data, such as observations of children’s behavior and data collected from multiple informants. If it is not feasible to do so, future work using parental reports on child outcomes should include a measure of parental social desirability as a covariate to strengthen the interpretability of findings and point to actual change in child outcomes as opposed to parent bias [105,106]. Future research would also benefit from additional follow-up assessments, as these would provide crucial insight into the long-term effects of MIIs, such as whether improvements in coping and perceptions of children’s behavior problems fade or continue to show lagged improvements, as has been found elsewhere [65].

Finally, it is worth noting that the mindfulness measure used in this study is not without criticism. Despite being one of the most commonly cited measures of mindfulness [107], some items tend to vary with meditation experience [108], indicating that the factor structure of the FFMQ may vary depending on sample. This ultimately calls into question what the true underlying structure of the FFMQ is. Others have criticized the measure for not being solely a measure of mindfulness, but also of more general processes that support being mindful [109]. Recently, some researchers have developed a single-item mindfulness item that may address concerns of construct validity and reliability across samples [110]. Future work in the measurement of mindfulness, including using the new single-item measure, is a must. 

## 5. Conclusions

This study pursued dual aims in a sample of families with elevated levels of risk: (1) to determine if there were patterns of cognitive coping styles and (2) evaluate the effectiveness of an MII in improving parents’ use of adaptive coping strategies and perceptions of children’s behavior among a sample with high risk. An oblimin-rotated PCA revealed three broad patterns of coping: *positive adaptation*, *negative adaptation*, and *positive refocusing*. Preliminary evidence suggests that a brief MII improves adaptive coping strategies and perceptions of children’s behavior over time. Ultimately, MIIs may be an accessible way to bolster family functioning in populations underrepresented in the mindfulness intervention literature. The study has important implications for both research and practice. Researchers should continue to probe family-level relationships to determine how a parent’s mindfulness benefits their children and potentially other family members. Practitioners can emphasize adaptive cognitive coping strategies. 

## Figures and Tables

**Table 1:** General themes of mindfulness intervention sessions.

Session	Cohort 1	Cohort 2
1	Mindfulness in the context of automatic habits	Mindful parenting and problem solving
2	Mindful reappraisal	Triggers of stress
3	Savoring positive experiences	Children’s needs
4	Triggers of stress	Stress reduction
5	Mindful parenting	Problem solving
6	Mindful planning	Mindful planning

**Table 2:** Item-factor loadings for the final three-factor solution of the CERQ-SF.

Item	Loadings		
	1	2	3
I think that I can become a stronger person because of what has happened	**0.785**	−0.166	−0.201
I think I can learn something from this situation	**0.750**	−0.142	−0.265
I think that I have to accept the situation	**0.575**	0.197	−0.455
I think about how to change the situation	**0.621**	0.342	0.141
I think about a plan of what I can do best	**0.681**	0.060	−0.169
I tell myself that there are worse things in life	**0.589**	−0.369	−0.040
I often think about how I feel about what I have experienced	0.343	**0.615**	0.149
I am preoccupied with what I think and feel about what I have experienced	−0.147	**0.723**	0.240
I keep thinking about how terrible it is what I have experienced	0.130	**0.817**	−0.034
I continually overthink how horrible the situation has been	−0.076	**0.816**	−0.222
I think of pleasant things that have nothing to do with it	0.520	−0.076	**0.670**
I think of something nice instead of what has happened	0.392	−0.055	**0.747**

Note: Extraction method = principal component analysis with a direct oblimin rotation; boldface indicates strongest item loadings retained for each factor using Comrey and Lee’s [100] cutoff of ≥0.55.

## Data Availability

Data are available from the first author upon request.
